# Academic Productivity at Orthopaedic Surgery Sports Medicine Fellowship Programs in the United States Has a Weak Positive Correlation With Nonresearch Lifetime Industry Earnings

**DOI:** 10.1016/j.asmr.2024.101042

**Published:** 2024-11-09

**Authors:** Benjamin Miltenberg, William L. Johns, Anthony N. Baumann, Faheem Pottayil, Bradley Richey, Albert T. Anastasio, Kempland C. Walley, Christopher C. Dodson, Sommer Hammoud

**Affiliations:** a Rothman Institute at Thomas Jefferson University, Philadelphia, Pennsylvania, U.S.A.; b College of Medicine, Northeast Ohio Medical University, Rootstown, Ohio, U.S.A.; c Medical College of Georgia, Augusta University, Augusta, Georgia, U.S.A.; d Department of Orthopedic Surgery, University of Michigan, Ann Arbor, Michigan, U.S.A.; e Department of Orthopedic Surgery, Duke University, Durham, North Carolina, U.S.A.

## Abstract

**Purpose:**

To characterize the relationship between academic productivity, as defined by the h-index, and industry payments for fellowship-trained sports medicine surgeons in faculty positions at sports medicine fellowships.

**Methods:**

The American Orthopaedic Society for Sports Medicine fellowship directory was used to create a comprehensive list of all fellowship programs nationwide. Fellowship websites were then reviewed to generate a list of the teaching faculty associated with each program. Total nonresearch lifetime earnings were obtained from the Centers for Medicare & Medicaid Services website. Academic productivity of each fellowship faculty was assessed via the h-index. Frequency counts and other descriptive statistic measures were used to describe the data for this study. Correlation was performed for continuous data using Spearman’s ρ.

**Results:**

Ninety orthopaedic surgery sports medicine fellowships were identified with a combined total of 574 orthopaedic surgery sports medicine fellowship faculty. There was a weak positive correlation between individual physician h-index and individual physician lifetime earnings at orthopaedic surgery sports medicine fellowships (*P* < .001; Spearman’s ρ = 0.329). There was a statistically significant difference between individual faculty h-index by quartile and individual faculty lifetime earnings (test statistic: 47.3; *P* < .001). There was no significant regional difference in payments, but there is remarkable heterogeneity in the distribution of payments to individual physicians, with the top 10% of physicians receiving over 80% of industry dollars.

**Conclusions:**

There is a positive correlation between academic productivity and industry payments at both the individual and institutional levels in orthopaedic sports medicine departments, although this relationship was greater at the fellowship level. Furthermore, the majority of nonresearch industry funding goes to a minority of physicians.

**Clinical Relevance:**

Evaluating the impact that nonresearch industry payments have on a sports medicine orthopaedic surgeon’s research productivity can offer valuable insights into the relationship between industry compensation and scholarly output in this field.

Introduced in 2013, the Physician Payments Sunshine Act (PPSA) created standards for a high degree of transparency regarding industry to physician payments.^1^^,^^2^ Incorporated as part of the Affordable Care Act, the PPSA requires that drug and device manufacturers disclose payments to physicians, shedding light on the complex relationship between physicians and industry. Orthopaedic surgeons receive and report funding at a disproportionately higher rate than other physicians.^1^ Payments to orthopaedic surgeons accounting for approximately 25% of the total payments made to all physicians and nearly 70% of orthopaedic surgeons have received payment from industry partners.^1^ While the American Orthopaedic Association and the Orthopedic Institute of Medicine highlighted the value of these relationships, they also emphasized the importance of transparency and ethical interactions with industry.^3^

Like any financial relationship, the connection between physicians and the medical device industry has the potential to impart bias, impacting many aspects of medicine, from research outcomes to clinical decision-making.^4^ In an effort to understand these relationships, there have been numerous studies analyzing these payments to highlight trends and patterns related to their distribution.^1^^,^^2^^,^^5^, ^6^, ^7^, ^8^, ^9^ While there has been extensive research addressing how industry payments impact practice patterns in orthopaedic sports medicine, less is known about what drives industry payments and which surgeons receive industry payments.^4^^,^^10^^,^^11^ Furthermore, while the impact that academic productivity has on the career of physicians has been studied, it is less clear if there is any correlation between industry payments to sports medicine surgeons and academic productivity.^12^, ^13^, ^14^, ^15^, ^16^ The purpose of this study was to characterize the relationship between academic productivity, as defined by the h-index, and industry payments for fellowship-trained sports medicine surgeons in faculty positions at sports medicine fellowships. We hypothesize that there is a positive correlation between research productivity and lifetime payments for fellowship-trained sports medicine surgeons and sports medicine fellowship programs.

## Methods

This study was designed as a retrospective analysis examining the relationship between academic productivity and industry payments of individual sports medicine surgeons and orthopaedic sports surgeons as of February 2024. While the Open Payments System identifies physicians by specialty, there is no subclassification for sports medicine. Thus, we turned to a directory created by the American Orthopaedic Society for Sports Medicine (AOSSM) and fellowship websites to generate a comprehensive list of sports medicine surgeons in faculty positions at accredited sports medicine fellowships. As of study creation in March 2023, the most recent AOSSM fellowship directory was used for fellowship information to create a comprehensive list of the fellowship programs to be analyzed. Each individual fellowship obtained from the AOSSM fellowship directory was then individually searched online for a list of surgical sports medicine faculty members. Data were collected from March 2023 to February 2024. All information used for this study is available for public access, and therefore, institutional review board approval for this study was not required.

### Eligibility Criteria

Inclusion criteria for this study were sports medicine surgeons with faculty positions at orthopaedic surgery sports medicine fellowships listed on the AOSSM directory with complete h-index and total lifetime industry earning information. Aggregate individual physician information for fellowship programs was included if at least 1 physician had complete data from that orthopaedic surgery sports medicine fellowship program. Individual physicians were excluded if they were missing an h-index on Scopus, total lifetime industry earnings on the Centers for Medicare & Medicaid Services (CMS) website, or both variables. Fellowships were excluded if no employed physician from that fellowship had complete information regarding their academic and industry productivity or if the fellowship list of employed physicians could not be found online.

### Study Variables

The main study variables were academic productivity and nonresearch industry income. Total nonresearch lifetime earnings were obtained from the CMS website on the Open Payments Database (OPD). OPD groups payment into the following categories: general payments, research payments, associated research funding, and ownership/investment interest. For the purpose of this study, nonresearch payments were calculated from the aggregation of payments in the general payments and ownership/investment interest categories. Thus, “total lifetime earnings” reflected nonresearch industry payments, such as royalties, consulting fees, food, lodging, and other payments not linked to research. For the purposes of this study, industry productivity for individual physicians was defined as their total nonresearch lifetime industry payments (US dollars). Industry productivity for fellowships was defined as the aggregated total lifetime payments (US dollars) of all physicians who comprise the fellowship faculty. Academic productivity was defined by the h-index as found on the Scopus website. The h-index is a metric that assesses academic productivity and impact by counting the number of publications of a researcher that has been cited at least as many times as the number of publications.^17^ Region of the United States (Northeast, Midwest, Southeast, and Southwest/West Coast) was also analyzed to describe the general geographic distribution of the fellowship programs throughout the United States. The Southwest and West Coast regions were combined due to a small sample size in the Southwest region.

### Data Extraction

Data extraction was performed by 2 authors (A.N.B. and F.P.). Data collected in the current study included h-index per individual faculty physician, total lifetime earnings per individual faculty physician, combined h-index per fellowship, combined total lifetime earnings per fellowship, and region of the United States. Data for each individual physician (h-index, lifetime earnings, etc.) were collected on the same date. Variables were recorded based on their value at the time of collection. To better analyze the relationship between total lifetime earnings and h-index, individual physicians and fellowships were placed in quartiles depending on their respective h-index scores, with the first quartile representing the lowest quartile of the h-index and the fourth quartile representing the highest quartile of the h-index. Data were taken from the “General Payments” category on the CMS website to represent nonresearch money directly paid to the physicians at the fellowship programs in this study.

### Statistical Considerations

This study utilized SPSS version 29.0 (IBM Corp) for analysis. Based on the large sample sizes, the Kolmogorov-Smirnov test was used to determine the normality of the data and the choice of statistical testing. Frequency counts and other descriptive statistic measures (median, mean with standard deviation, minimum, and maximum) were used to describe the data for this study. Medians between 3 or more groups for subgroup analysis were compared using the independent samples median test with Bonferroni correction for post hoc testing as the data were nonparametric and contained extreme outliers for each set. Correlation was performed for continuous data using Spearman’s ρ due to the nonparametric distribution of the data. Significance values were set at *P* < .05 for all analyses undertaken.

## Results

### Initial Search Results

The initial search resulted in a total of 90 orthopaedic surgery sports medicine fellowships in the United States, with a combined total of 574 orthopaedic surgery sports medicine fellowship faculty. After excluding fellowships and physicians without complete CMS financial data and h-index data, there were 84 fellowships (93.3%) and 539 individual physicians (93.9%) included for analysis.

### Individual Physician Demographics

The median individual physician (n = 539 physicians) lifetime earnings reported on the CMS website was $25,571.55 (mean: $319,347.60 ± $2,499,116.73; minimum-maximum: $0.00-$52,150,069.08) (Table 1). Median individual physician (n = 539 physicians) h-index as reported on Scopus was 13.0 (mean: 17.65 ± 16.70; minimum-maximum: 0.0-91.0).

**Table 1:** Information on Faculty Physicians at Orthopaedic Surgery Sports Medicine Fellowships in the United States With the Top 5 Total Lifetime Earnings

Individual Physician Ranking by Total Lifetime Earnings	Individual Physician H-Index	Individual Physician Total Lifetime Earnings
1	43	$52,150,069.08
2	60	$22,543,620.02
3	13	$6,282,391.89
4	61	$5,121,630.01
5	70	$4,343,863.04

NOTE. Information includes individual physician h-index and individual physician total lifetime earnings.

### Academic and Industry Productivity for Individual Physicians

**Fig 1:**
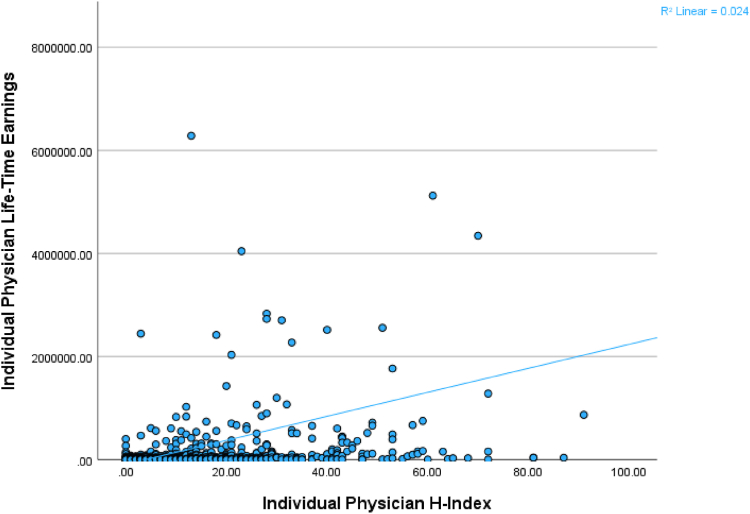
The correlation between individual faculty h-index and individual physician lifetime earnings associated with orthopaedic surgery sports medicine fellowships. The y-axis was set at $8,000,000 to allow for better representation of the relationship of the data. Line represents best-fit line for correlation with a weak positive correlation between the h-index and lifetime earnings for individual physicians.

There was a weak positive correlation between individual physician h-index and individual physician lifetime earnings at orthopaedic surgery sports medicine fellowships (*P* < .001; Spearman’s ρ = 0.329) (Fig 1). There was a statistically significant difference between individual faculty h-index by quartile and individual faculty lifetime earnings (test statistic: 47.3; *P* < .001). The fourth quartile by h-index (n = 134 physicians; median h-index: 39.5; median total lifetime earnings: $97,572.47) had significantly more total lifetime earnings than the first quartile (*P* < .001), the second quartile (*P* < .001), and the third quartile (*P* = .001).

### Industry Productivity Distribution for Individual Physicians

Aggregating the 539 individual physicians included in this study, there was a total of $172,128,356.50 in nonresearch lifetime earnings reported on the CMS website. The top 10% earning faculty at orthopaedic surgery sports medicine fellowships (n = 54 physicians) received 84.7% of the nonresearch lifetime industry earnings ($145,874,208.70 out of a total $172,128,356.50), whereas the top 1% of physicians (n = 5 physicians) received 52.5% of the nonresearch lifetime earnings ($90,441,574.04 out of a total $172,128,356.50).

### Fellowship Demographics

The median combined physician h-index per fellowship (n = 84 fellowships) was 100.5 (mean: 113.3 ± 79.5; minimum-maximum: 3.0 - 365.0) (Table 2). The median combined physician lifetime earnings per fellowship was $452,700.56 (mean: $2,049,147.10 ± $6,685,146.31; minimum-maximum: $6,074.52-$53,670,641.85).

**Table 2:** Top 10 Orthopaedic Surgery Sports Medicine Fellowships in the United States by Combined Fellowship Total Lifetime Earnings

Fellowship Ranking by Total Lifetime Earnings	Fellowship H-Index	Fellowship Total Lifetime Earnings	Program Type
1	179	$53,670,641.85	Private
2	259	$29,663,154.42	Private
3	365	$7,623,364.01	Mixed
4	54	$7,418,637.25	University
5	146	$6,129,267.61	Private
6	105	$5,261,998.48	Private
7	122	$4,741,951.97	Mixed
8	189	$4,042,513.17	University
9	272	$3,108,363.87	University
10	187	$2,919,552.62	University

NOTE. Table includes total lifetime earnings per fellowship, total h-index per fellowship, and program type for each fellowship.

### Academic and Industry Productivity for Fellowship Programs

**Fig 2:**
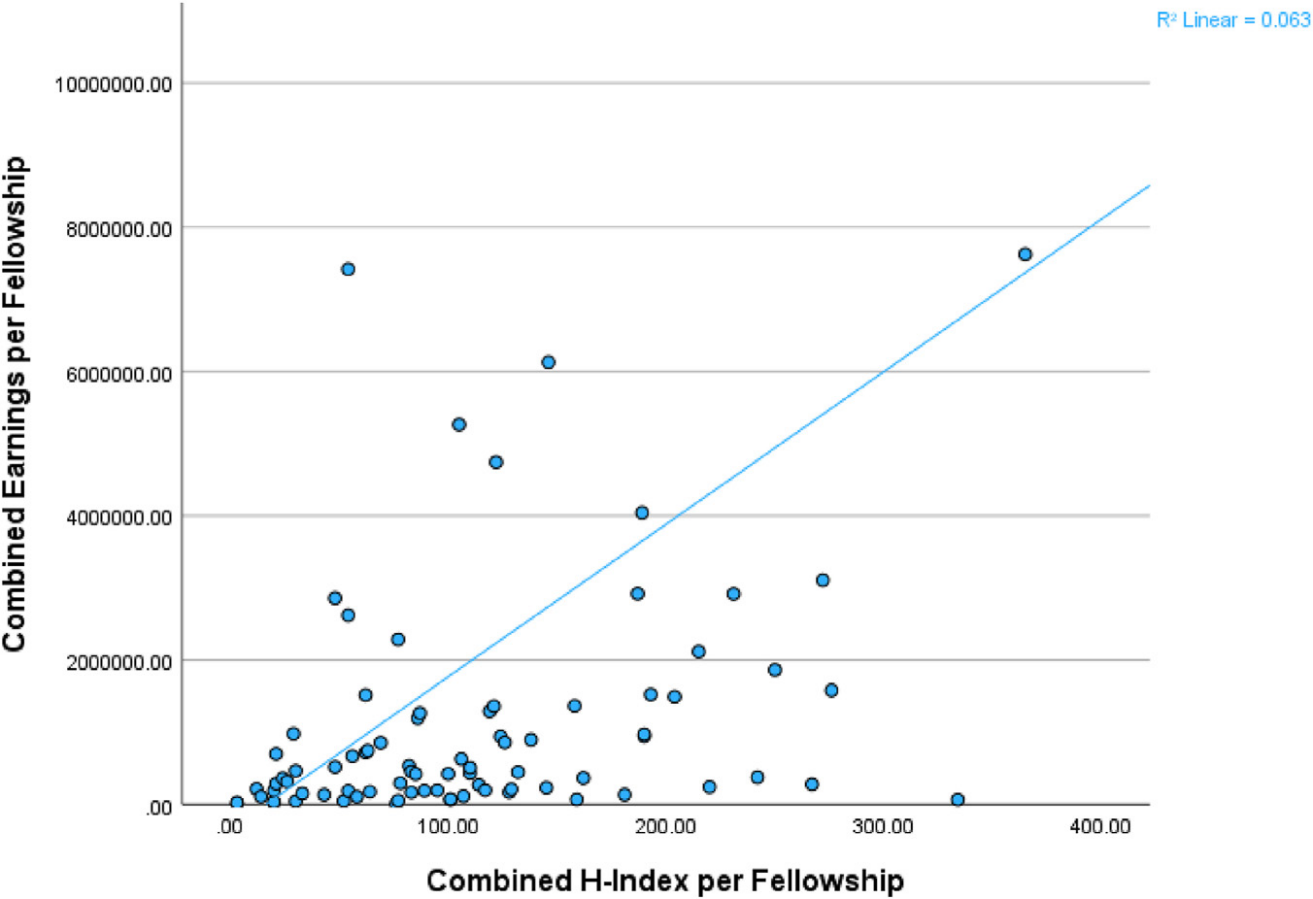
Correlation between the combined h-index per orthopaedic surgery sports medicine fellowship and the combined earnings reported on the Centers for Medicare & Medicaid Services website. The y-axis was set at $10,000,000 to allow for better representation of the relationship of the data. Line represents best-fit line for the correlation and demonstrates moderate positive correlation.

There was a moderate positive correlation between combined fellowship h-index and total lifetime earnings per fellowship at orthopaedic surgery sports medicine fellowships (*P* < .001, Spearman’s ρ = 0.432) (Fig 2). Overall, there was no statistically significant difference between combined fellowship h-index by quartile and total lifetime earnings per fellowship (test statistic: 6.286; *P* = .099). However, on post hoc testing, only the fourth quartile by h-index (n = 21 fellowships; median h-index: 215.0; median total lifetime earnings: $1,523,411.65) had significantly more total lifetime earnings than the first quartile (n = 21 fellowships; median h-index: 26.0; median total lifetime earnings: $190,622.48) (*P* = .033) with no statistically significant difference in total lifetime earnings when compared to the second quartile (n = 21 fellowships; median h-index: 77.0; median total lifetime earnings: $456,372.06) (*P* = .185) and third quartile (n = 21 fellowships; median h-index: 121.0; median total lifetime earnings: $507,880.05) (*P* = .185).

### Academic and Industry Productivity for Fellowship Programs by Region

There was no statistically significant difference in total lifetime earnings per fellowship when stratified by region of the fellowship program in the United States (test statistic: 2.759; *P* = .430) (Table 3). Furthermore, there was no statistically significant difference in h-index per fellowship when stratified by region of the fellowship program in the United States (test statistic: 5.693; *P* = .128). After post hoc testing, none of the regions (Northeast, Midwest, Southeast, or Southwest/West Coast) had any statistically significant differences in total lifetime earnings or h-index compared to the other regions.

**Table 3:** Information on the Total Lifetime Earnings and H-Index per Fellowship by Region of the United States

Region	Number of Fellowships	Median H-Index per Fellowship	Median Total Lifetime Earnings per Fellowship
Northeast	18	125.0	$584,136.05
Midwest	27	106.0	$433,340.04
Southeast	24	82.5	$526,298.63
Southwest/West Coast	15	77.0	$287,066.47

NOTE. Data include region, number of fellowships per region, median h-index per fellowship, and median total lifetime earnings per fellowship.

## Discussion

The main finding of this study is that there is a positive correlation between academic productivity and industry payments at both the individual and institutional levels in orthopaedic sports medicine departments. Importantly, the weak and positive correlation (Spearman’s ρ = 0.329) on an individual level improved to a moderate and positive correlation (Spearman’s ρ = 0.432) when examining this relationship for fellowships as a whole (rather than for individual faculty members). Furthermore, there was no significant regional difference in industry earnings, but there was remarkable heterogeneity in the distribution of payments to individual physicians, with the top 10% of physicians receiving over 80% of industry dollars.

While our data suggest a connection between intellectual labor and industry compensation, the relationship may not be apparent to patients. Trust and credibility are vital to the surgeon-patient relationship and may be affected by financial ties to industry. This belief may contribute to the finding of Hughes et al.,^18^ who found that many sports medicine surgeons fail to reveal industry support of any kind. Furthermore, a systematic review of the impact of industry financial ties to the patient-physician relationship found that patients believe a financial connection to industry decreases the quality of the care they receive.^19^ Despite this, however, Camp et al.^20^ found that in orthopaedics, patients generally approve of financial relationships between surgeons and industry that support innovation and research. In light of the findings of Camp et al.^20^ and the information presented in this study, surgeons who participate in industry must respect that the surgeon-industry relationship is a delicate balance. Surgeons may find it beneficial to discuss the connection between the innovation vital to our field and financial relationships between surgeons and industry.

While we found a positive correlation between academic productivity and industry-driven compensation, prior literature shows an inconsistent relationship between these 2 variables. Buerba et al.^5^ found that when looking at all orthopaedic subspecialties, higher mean h-indexes and number of publications were weakly correlated with nonresearch payments. In contrast, Chen et al.^6^ found that while the surgeons who receive industry research payments had significantly higher h-indexes, there was no significant difference in h-index between surgeons receiving nonresearch funding and those not receiving nonresearch funding. However, they did show a positive correlation between the amount of nonresearch funding and h-index of surgeons.^6^ While on the surface, this may seem contradictory, it may be explained by the volume of orthopaedic surgeons who receive funding in general and the diversity in payment dollars between those surgeons. A substantial majority of orthopaedic surgeons receive industry funding, and by law, all payment/benefits/reimbursement over 10 dollars must be reported.^1^^,^^2^ Thus, there is a large continuum in compensation between surgeons. Therefore, grouping all surgeons who receive any industry payment into a single group creates a categorical variable out of data that might be more accurately represented as a continuous variable. For instance, a surgeon who was compensated 10 dollars would be considered the same as a surgeon who was compensated 1 million dollars, which could help to explain why Chen et al.^6^ found no significant difference in h-index between surgeons receiving and not receiving nonresearch funding, but there was a positive correlation between h-index and total dollars of industry nonresearch funding.

Interestingly, we found that industry payments are neither evenly nor normally distributed among surgeons. The top 10% of faculty at surgery sports medicine fellowships receive 84.7% of the nonresearch payments, and the top 1% of faculty at surgery sports medicine fellowships receive 52.5% of the nonresearch payments. These findings are unsurprising and remain consistent with prior literature. In 2015, Samuel et al.^7^ found that orthopaedic surgeons have a notably high Gini coefficient (0.959), indicating a generally large disparity in industry payments within the field of orthopaedics. Moreover, they found this disparity to be higher in orthopaedics than other medical specialties.^7^ Thus, it is unsurprising that in 2021, Partan et al.^21^ found that within the field of sports medicine, the top 5 compensated surgeons received 45.8% of all industry contributions, and most of those dollars were attributed to royalties and licenses (98.7%). To this effect, our study confirms what has been previously established—namely, that most industry dollars are given to a very small minority of surgeons. When analyzing these trends across orthopaedic subspecialties, in similarly designed analyses of PPSA-reported payments, orthopaedic spine surgeons and spine fellowship programs shared similar correlations between academic productivity and nonresearch industry earnings to that reported in the present study of sports medicine surgeons.^8^ In contrast, however, foot and ankle surgeons and programs showed no correlation.^9^ The reason for these findings are likely multifactorial, but there were significantly fewer foot and ankle faculty (165 physicians) and fellowship programs (48) than sports medicine (574 faculty, 90 programs) and spine (320 faculty, 75 programs) programs reviewed, and thus, a lack of sufficient power to detect a meaningful correlation could be the primary cause.^8^^,^^9^^,^^18^^,^^22^ These findings suggest industry-based financial compensation is directed to sports medicine surgeons who have a proportionately larger role in advancing the field of sports medicine through impactful academic publications. Innovation is a critical component of any field of medicine, fostering the improvements in patient care that drive our profession forward. Innovation and research are undoubtedly linked, making physicians engaged in research ideal candidates for involvement with medical innovation. This connection may help drive the relationship between research productivity and industry compensation—compensation that is predominantly driven by royalty fees. While any financial connection to industry has the potential to impart bias,^18^^,^^22^ it is reassuring to know this financial relationship is at least in part driven by physicians’ contributions to innovation in the field. This connection could also be driven by the financial freedom provided by industry payments; it is possible that money provided by industry provides physicians the financial security needed to pursue academic publications instead of further expanding their clinical practice. Another possible reason for this connection may be due to the fact that physicians who are engaged academically are more exposed to industry and thus are more likely to be engaged with industry financially. Additionally, surgeons linked to fellowships may be more pursued by industry, given their role in teaching other surgeons and the impact that may have on future surgeons’ implant/device choices. Future research may help to tease apart this relationship by comparing industry payment between surgeons associated with fellowships/residences and those not affiliated with surgical education.

### Limitations

Our study must be interpreted within the context of its limitations. First and foremost, we used the h-index as a proxy for academic productivity. Although the h-index is widely used in this capacity, it remains subject to inherent bias as it may be artificially inflated for surgeons with longer careers, which naturally increases citation count.^17^^,^^23^ We were unable to control for age/career length as this information was not readily accessible, and thus the positive correlation between h-index and payments could represent a relationship between experience and industry funding. Additionally, we relied on the OPD to account for funding information. This database was created by submissions from industry, placing the data at risk of reporting bias. Additionally, there could conceivably be an advantage for larger fellowship programs with more faculty, which would theoretically result in larger overall combined payments and h-indices. However, our main study comparison is a continuous correlation between the h-index and career earnings, which should adequately control for varying program sizes. Additionally, however, there could be discrepancies between the lifetime earnings of surgeons and their time of affiliation with a given fellowship. Also, our analysis of academic productivity and industry payments based on region may be limited by our allocation within only 5 regions throughout the United States rather than a more granular inspection. Finally, we were unable to assess how sex impacts industry compensation. This is because coding for this variable would have required us to make an assumption about the physician’s sex based on name and photograph.

## Conclusions

There is a positive correlation between academic productivity and industry payments at both the individual and institutional levels in orthopaedic sports medicine departments, although this relationship was greater at the fellowship level. Furthermore, most nonresearch industry funding goes to a minority of physicians.

## Disclosures

The authors declare the following financial interests/personal relationships which may be considered as potential competing interests: C.D.C. reports a relationship with aevumed that includes consulting or advisory and equity or stocks. S.H. reports a relationship with Arthrex that includes consulting or advisory and speaking and lecture fees. All other authors (B.M., W.L.J., A.N.B., F.P., B.R., A.T.A., K.C.W.) declare that they have no known competing financial interests or personal relationships that could have appeared to influence the work reported in this paper.
